# Enhanced Transdermal Permeability via Constructing the Porous Structure of Poloxamer-Based Hydrogel

**DOI:** 10.3390/polym8110406

**Published:** 2016-11-21

**Authors:** Wen-Yi Wang, Patrick C. L. Hui, Elaine Wat, Frency S. F. Ng, Chi-Wai Kan, Clara B. S. Lau, Ping-Chung Leung

**Affiliations:** 1 Institute of Textiles and Clothing, The Hong Kong Polytechnic University, Hung Hom, Kowloon, Hong Kong, China; 13901847r@connect.polyu.hk (W.-Y.W.); tchuip@polyu.edu.hk (P.C.L.H.); frency.ng@polyu.edu.hk (F.S.F.N.); 2 Institute of Chinese Medicine, The Chinese University of Hong Kong, Shatin, New Territories, Hong Kong, China; elaine.wat.cuhk@gmail.com (E.W.); claralau@cuhk.edu.hk (C.B.S.L.); pingcleung@cuhk.edu.hk (P.-C.L.); 3 State Key Laboratory of Phytochemistry and Plant Resources in West China, The Chinese University of Hong Kong, Shatin, New Territories, Hong Kong, China

**Keywords:** enhanced permeability, hydrogel matrix, porous structure, transdermal drug delivery

## Abstract

A major concern for transdermal drug delivery systems is the low bioavailability of targeted drugs primarily caused by the skin’s barrier function. The resistance to the carrier matrix for the diffusion and transport of drugs, however, is routinely ignored. This study reports a promising and attractive approach to reducing the resistance to drug transport in the carrier matrix, to enhance drug permeability and bioavailability via enhanced concentration-gradient of the driving force for transdermal purposes. This approach simply optimizes and reconstructs the porous channel structure of the carrier matrix, namely, poloxamer 407 (P407)-based hydrogel matrix blended with carboxymethyl cellulose sodium (CMCs). Addition of CMCs was found to distinctly improve the porous structure of the P407 matrix. The pore size approximated to normal distribution as CMCs were added and the fraction of pore number was increased by over tenfold. Transdermal studies showed that P407/CMCs saw a significant increase in drug permeability across the skin. This suggests that P407/CMC with improved porous structure exhibits a feasible and promising way for the development of transdermal therapy with high permeability and bioavailability, thereby avoiding or reducing use of any chemical enhancers.

## 1. Introduction

The transdermal drug delivery system has made considerable progress since the first transdermal drug patch that delivers scopolamine to treat motion sickness was approved for use in 1979 [1]. Compared to oral delivery and hypodermic injection, transdermal delivery is considered an attractive candidate for drug delivery due to several advantageous features, e.g., avoidance of first-pass hepatic metabolism, prolonged period of drug release and superior patient compliance [2,3]. Significant advances in transdermal drug delivery systems have been made over the last three decades and delivery of drugs, including small-molecules drugs, macromolecules (peptides or deoxyribonucleic acid (DNA) and virus-based and other vaccines can now be made through targeted permeabilization by overcoming the skin’s barrier [1].

The major challenge in transdermal drug delivery consists in the skin’s outermost layer, namely, stratum corneum, which is 10–20 μm thick and has an organized structure [4]. To enhance the skin permeability, the most commonly used approach is the application of chemical penetration enhancers to enable the skin to be permeable to the desired drugs by overcoming the skin’s barrier effect. Penetration enhancers are normally incorporated into a carrier or formulation to permeabilize the skin and thus to improve the diffusivity of the drugs. However, this approach may be problematic because of the induced irritation or toxicity caused to living cells in the deeper skin [1]. Therefore, a wide variety of enhancement candidates have also been proposed and developed, including microneedle array [5], ultrasound [6], iontophoresis [7] and electroporation [8].

**Figure 1:**
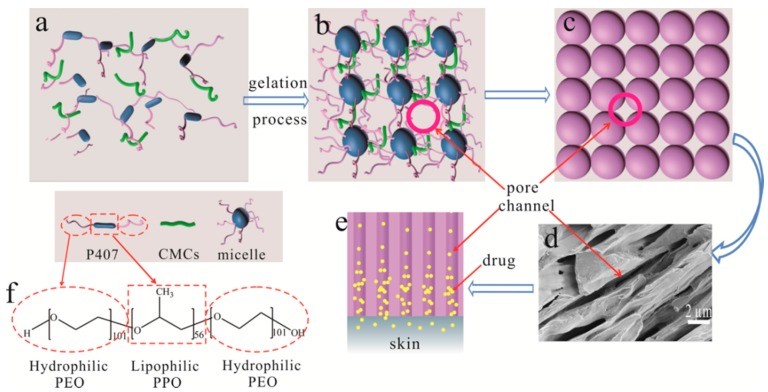
Schematic diagram to show the formation of pore channels based on poloxamer 407 (P407), (**a**) P407 molecule in sol state; (**b**) formation of micelles in gel state; (**c**) close-packed array model of micelles and formation of pore channels; (**d**) field emission scanning electron microscope (FE-SEM) image of sectional view of pore channels (×8k); (**e**) transport of drugs in the pore channels; and (**f**) hydrophilic poly(ethylene oxide) (PEO) chain and lipophilic poly(propylene oxide (PPO) chain of P407 molecule.

Additionally, more efforts have been made to develop new transdermal techniques and construct new formulation systems, such as smart hydrogel matrix [9] and novel vesicular systems, e.g., liposomes [10], ethosomes [11] and transferosomes [12]. To the best of our knowledge, however, the progress in the transdermal techniques has been focused only on breaking the skin’s barrier function, neglecting the resistance of carrier matrix for diffusion of drugs [4]. Actually, the diffusion and transport of drugs are always ineluctably regulated and impeded by the formulation matrix. Unfortunately, less attention has been paid to optimization and design of the drug formulation matrix to increase the drug concentration-gradient driving force and thus to enhance the diffusivity of drugs. This has motivated us to attempt to develop a novel drug formulation matrix with a porous channel structure, in which drugs can be entrapped and transported to a targeted location, to increase the percutaneous diffusional release and bioavailability of drugs therein for transdermal purposes, thereby avoiding or reducing the use of any chemical enhancers (Figure 1).

Hydrogels are hydrophilic three-dimensional polymeric networks, which can absorb a large amount of water or biological fluids [13]. The high moisture content renders hydrogels compatible with most living tissues and thus facilitates widespread application in biomedical and pharmaceutical areas [14,15]. Temperature responsive hydrogel exhibits a free-flowing sol at low temperatures, but becomes a gel at body temperature, which facilitates administration and accessibility when applied in drug delivery systems [16,17]. Poloxamer 407 (P407) is one of the most typical thermosensitive polymers, which consists of a triblock copolymer with amphiphilic structure (hydrophilic ethylene oxide moiety and hydrophobic propylene oxide moiety) and provides an excellent candidate to develop such a formulation [18,19]. P407 molecule can self-assemble into spherical micelles and shows strong temperature dependence in aqueous solutions, i.e., thermal sensitivity [20,21]. Association of the packed spherical micelles results in not only the formation of gel but also the creation of 3D cubic lattice (Figure 1) [18,22]. Accordingly, abundant cavity, i.e., porous channels, can be formed, through which the hydrophilic drugs can be transported and a concentration-gradient driving force can be produced due to aggregation of drug molecules. The obstruction of hydrogel matrix may thus be reduced, thereby increasing the percutaneous behavior of drugs and avoiding the use of any penetration enhancers. Furthermore, the thermo-sensitivity endows poloxamer-based formulation with sol-gel transition as the temperature changes, which facilitates administration and accessibility when applied in drug delivery systems [23]. The present study aims to establish the porous channels of poloxamer-based hydrogel formulation and examine its enhancement capacity for the targeted drugs in a transdermal delivery system. Hydrophilic carboxymethyl cellulose sodium (CMCs) was used to reinforce the porous channels and regulate the physicochemical properties of poloxamer matrix.

## 2. Materials and Methods

### 2.1. Materials

Poloxamer 407 (P407, *M*_W_ = 11,500, PEO101-PPO56-PEO101) and carboxymethyl cellulose sodium (CMCs) were obtained from International Laboratory (South San Francisco, CA, USA) and Wako Chemicals (Wako, Japan), respectively. Dulbecco’s phosphate-buffered saline (DPBS) was provided by Thermo Fisher Scientific (Waltham, MA, USA). Cortex Moutan (CM) aqueous extract was a gift from the Institute of Chinese Medicine (ICM), the Chinese University of Hong Kong. Water-soluble azone (laurocapram) was purchased from Guangzhou Nanjia Chemical Technology Co. Ltd. (Guangzhou, China). All other reagents were of analytical grade and used as needed.

### 2.2. Preparation of P407/CMCs Composite Hydrogel

P407/CMCs composite hydrogel was fabricated using “cold method” as described by Schmolka [24]. Briefly, the weighed P407 was added into chilled water (<10 °C) with the final concentration at 16%, 20% and 24% (*w*/*w*) under constant magnetic stirring. The dispersion was then refrigerated until P407 was completely dissolved. The three batches were labeled as PC160, PC200 and PC240. P407/CMCs composite hydrogel was fabricated simply by adding CMCs into P407 solution (20%, *w*/*w*) under constant stirring with the final CMCs concentrations at 2% and 4% (*w*/*w*) labeled as PC202 and PC204, respectively. The fabrication of CM loaded composite hydrogel was similar, simply by dissolving P407 in CM solution, and the concentration of CM was 15% (*w*/*w*). The concentration of azone in the samples involved was 3% (*w*/*w*).

### 2.3. Viscosity Analysis

Viscosity of P407/CMCs hydrogel was analyzed using a Physica MCR301 rheometer (Anton Paar, Graz, Austria), operated with parallel plates (diameter 25 mm). Silicon oil was used to avoid water evaporation on the outer edge of the sample. The testing temperature ranged from 5 to 40 °C with a constant heating rate of 1 °C/min at a fixed frequency of 1 Hz. The curves of complex viscosity (η*) versus temperature were plotted, the inflection points of which were indicative of the gelation transition temperature (*T*_gel_).

### 2.4. Observation of Porous Structure

The porous structure of vacuum lyophilized P407/CMCs hydrogel was visually observed by field emission scanning electron microscope (FE-SEM). The samples were fixed with conductive tape on a metal stub, sputter coated with gold under vacuum and observed at a 3 kV accelerating voltage at a working distance of 8.0 mm using a JEOL 6490 (Hitachi, Tokyo, Japan).

### 2.5. Porosity Analysis

The most probable pore size, total surface area, pore number fraction and pore size distribution of vacuum lyophilized P407/CMCs composite hydrogel were determined by mercury intrusion porosimetry (Quantachrome PoreMaster 60, Quantachrome Instruments, Boynton Beach, FL, USA). Around 0.2 g of each sample was placed into the sample holder cell and was penetrated by mercury at high pressure. The data were obtained using Quantachrome PoreMaster for Windows, version 8.01 (Microsoft, Redmond, Washington DC, USA).

### 2.6. Transdermal Behaviour Analysis

Effects of the porous structure of P407/CMCs composite hydrogel on the drug’s percutaneous behavior were comparatively analyzed using a Franz diffusion cell (receptor compartment: 6.5 mL). The porcine ear skin was selected as permeation membrane to evaluate the drug’s percutaneous behavior. Fresh skin was excised and isolated from the porcine ears (purchased from a local butcher) with a scalpel. The excised ear skin was then placed between the donor and the receptor compartment of the diffusion cell. The samples were positioned over the skin and covered with paraffin film. The receptor compartment of the diffusion cell was filled with DPBS (pH 7.4) with temperature at 37 ± 0.5 °C and magnetic agitation at 300 rpm. Two milliliters of the samples were withdrawn at each time interval ranging from 24 to 48 h and replenished immediately with an equal volume of fresh DPBS to maintain sink condition. One of the principal active ingredients of CM, gallic acid (GA) permeated across the skin was assayed by a high-performance liquid chromatograph (HPLC, Hewlett Packard Agilent 1000 series, Hewlett-Packard, San Diego, CA, USA) [25]. All the experiments met the sink conditions and were repeated thrice.

The drug’s percutaneous behavior was studied by linear regression analysis. The apparent permeability coefficient (*P*_app_, cm/s) was calculated according to Equation (1) [26,27].

*P*_app_ = (*V*/(*A* × *Ci*)) × (d*Ca*/d*t*) (1)

where:

*V*: the volume of the receiving chamber (6.5 cm^3^);
*A*: the area of the skin surface exposed to the receiving chamber (2.8 cm^2^);
*Ci*: the initial concentration of GA in the hydrogel formulation (μg/cm^3^);
d*Ca*/d*t*: the change in the concentration of GA in the receiving chamber.

Based on the *P*_app_ value, to compare hydrogel Formulations (2) and (1), the enhancement ratio (*ER*) of drug permeation across the porcine skin was calculated according to Equation (2).

*ER* = *P*_app_ (2)/*P*_app_ (1) (2)

### 2.7. Statistical Analysis

The experimental data were analyzed using Origin software (version 9.0, OriginLab, Northampton, MA, USA), to obtain standard deviation, one-way Analysis of Variance (ANOVA) test and Bonferroni test. A *p*-value of 0.05 was considered as the required level of significance and the data were labeled with an asterisk (*) for *p* < 0.05, (**) *p* < 0.01. Each experiment was conducted thrice (*n* = 3).

## 3. Results

### 3.1. Viscosity Analysis

**Figure 2:**
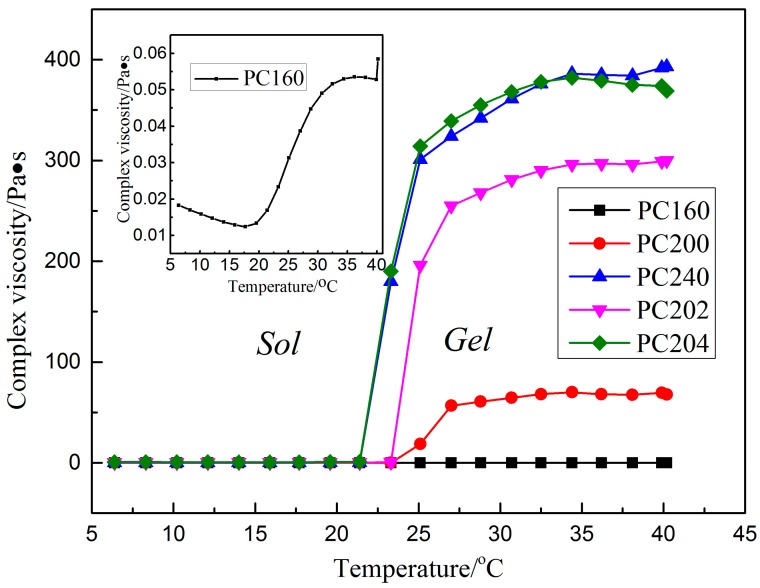
Complex viscosity versus temperature curves of various formulations of P407/carboxymethyl cellulose sodium (CMCs) hydrogel matrix (the data for PC200, PC202 and PC204 were extracted from references [28,29] for the sake of argument).

Rheological properties of P407 matrix with varying concentrations of 16%, 20% and 24% (*w*/*w*) and P407/CMCs composite hydrogel matrix have been discussed in our previous work [28,29]. The present study extracted and focused on the viscosity data in order to facilitate further discussion (Figure 2). For P407 matrix, as expected, viscosity was found to be positively associated with the matrix concentration. PC160 saw the lowest complex viscosity above the critical gelation temperature, while the figure for PC200 was slightly higher. Conversely, a sharp increase was observed in PC240 in the complex viscosity. Meanwhile, the sol-gel transition temperature shows a similar trend, i.e., an appreciable decline from around 23 °C for PC160 and PC200 to 21 °C for PC240.

Based on the viscosity of P407 matrix, concentration of 20% (*w*/*w*) was selected for further study. As can be seen from Figure 2, by comparing the samples of PC200, PC202 and PC204, the complex viscosity shows a significant increase with an increase in the proportion of CMCs. However, the gelation transition temperature appears to have an opposite trend, a drop from around 23 °C for PC200 and PC202 to 21 °C for PC204. It should be noted that the complex viscosity for PC204 was comparable to that of PC240.

### 3.2. Porosity Analysis for P407 Matrix

**Figure 3:**
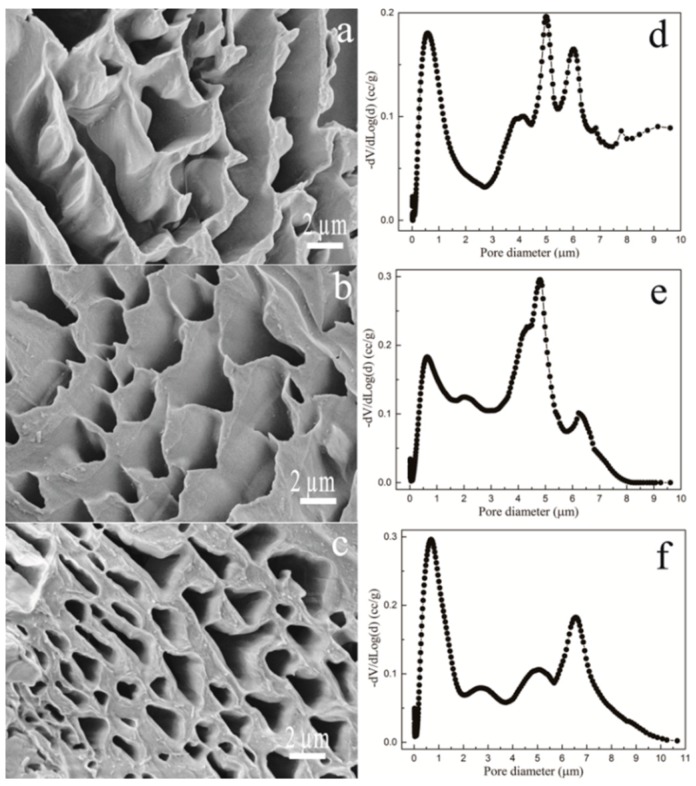
Pore surface morphology of P407 matrix hydrogel, PC160 (**a**) (5000×); PC200 (**b**) (5000×) and PC240 (**c**) (5000×); and the corresponding pore size distribution for PC160 (**d**); PC200 (**e**) and PC240 (**f**).

The pore morphology of P407 matrix with varied concentrations is shown in Figure 3a–c. FE-SEM images indicate that P407 matrix presents an abundantly porous structure, which was shown to be visually concentration dependent. It can be observed that P407 with a low concentration tends to produce large pores. The pore size and distribution were further quantitatively analyzed (Figure 3d–f and Table 1). As shown in Figure 3d–f, the pore size for P407 matrix shows a broad distribution, ranging from around 0.2 to 10 μm. Table 1 presents the most probable pore size and the number fraction. The most probable pore size for PC160 is slightly higher than that of PC200, whereas PC240 saw a sharp decline, at 0.689 μm. The pore number fractions for the three samples were comparable, without a statistically significant difference.

### 3.3. Porosity Analysis for P407/CMCs

**Figure 4:**
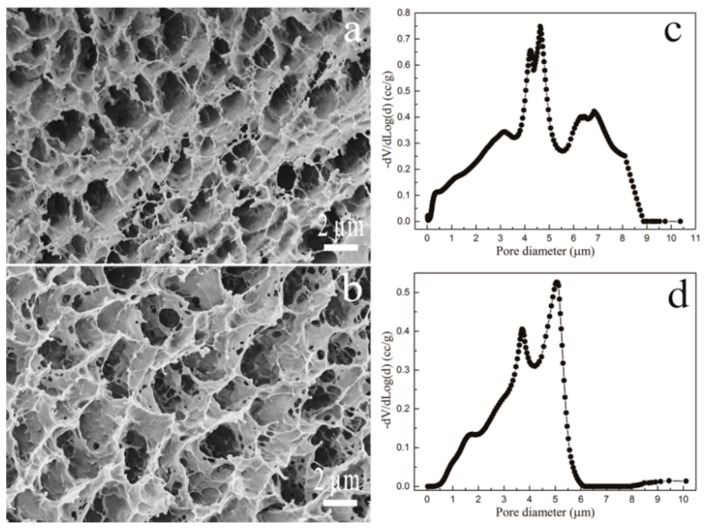
Pore morphology of P407/CMCs hydrogels of PC202 (**a**) (5000×) and PC204 (**b**) (5000×); and the corresponding pore size distribution of PC202 (**c**) and PC204 (**d**).

The surface morphology for the porous structure of P407/CMCs composite matrix (Figure 4a,b) shows a significant change in the pore shape and size, with the presence of CMCs. The quantitative analysis (Figure 4c,d) indicated that the pore size distribution is close to normal distribution, both for PC202 and PC204. Compared to PC202, PC204 exhibits a slightly narrow distribution range. This observation was consistent with the pore number fraction (Table 1), which was the highest for PC204. This indicates that the porous structure of P407 matrix was significantly optimized and improved with the presence of CMCs, and the concentration of 4% (*w*/*w*) CMCs gave rise to the optimal pore size distribution.

### 3.4. Percutaneous Analysis

**Figure 5:**
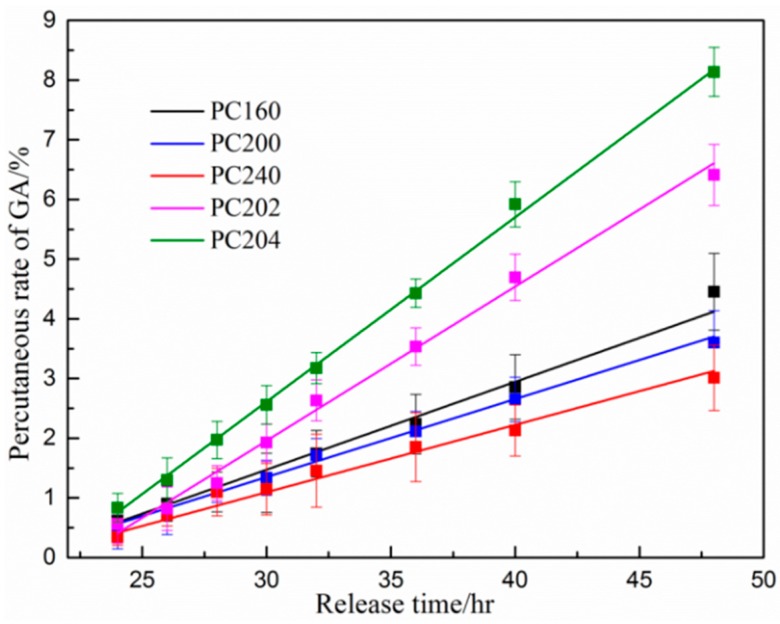
In vitro cumulative diffusional release of gallic acid (GA) from various P407/CMCs hydrogel matrix penetrated through porcine ear skin (*n* = 3) (HPLC analysis: mobile phase consisting of acetonitrile and double distilled water/phosphoric acid (99.0/1.0, *v*/*v*); flow rate: 1.0 mL/min) (the data for PC200, PC202 and PC204 were extracted from reference [29] for the sake of argument).

**Figure 6:**
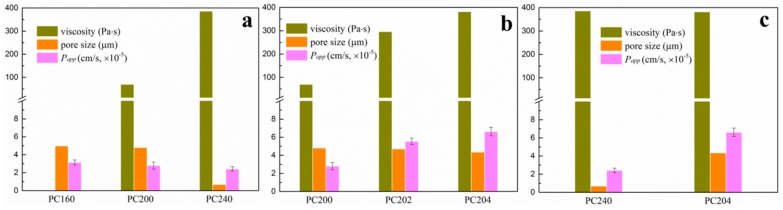
To facilitate discussion, various P407/CMCs composite hydrogels were grouped to comparatively analyze their complex viscosity, the most probable pore size and apparent permeability coefficient: (**a**) shows PC160, PC200 and PC240; (**b**) shows samples with different CMCs content, i.e., PC200, PC202 and PC204; and (**c**) compares samples with comparable viscosity: PC240 and PC204. (Viscosity data extracted at 37 °C).

The percutaneous diffusional behavior of the model drug from various P407 based hydrogel matrix was investigated by analyzing the concentration of GA penetrated through porcine ear skin. The concentration of GA in the receiving chamber was determined 24 h after release in order to make the accumulated GA detectable for HPLC analysis. As shown in Figure 5, the accumulative penetration percentage of GA was linearly correlated with the diffusional release time. However, there were significant differences among the percutaneous diffusion of GA from the various P407/CMCs hydrogel matrix (*p* < 0.05). By comparing the P407 matrix with different concentrations, i.e., PC160, PC200 and PC240, matrix concentration showed an appreciable influence on the percutaneous diffusivity for the model drug. A higher P407 concentration tends to result in a lower GA penetration level. This is in good agreement with the apparent permeability coefficients (*P*_app_ value) shown in Figure 6a, where PC160 had the highest *P*_app_ value among the three specimens. With the presence of CMCs, both PC202 and PC204 saw a considerable increase in the transdermal diffusivity and showed higher *P*_app_ values (Figure 6b). By contrast, PC204 had the highest *P*_app_ values and showed the optimal matrix formulation in transdermal diffusivity.

## 4. Discussion

Transdermal drug delivery provides an attractive alternative to oral delivery and hypodermic injection due to several advantageous traits, such as prolonged period of steady drug delivery, avoiding hepatic first-pass effect and good patient compliance [1,30,31]. The drug in the transdermal delivery system is typically entrapped and regulated in a polymeric carrier formulation, such as nanoemulsions [32], vesicles [33], nanoparticles [34] and polymeric gel [35], etc. Accordingly, in addition to the skin’s barrier effect, another challenge for diffusion and transport of targeted drugs primarily stems from the resistance of the carrier matrix. This provides an explanation for the fact that transdermal drug delivery systems always have a low bioavailability and a wide variety of penetration enhancing approaches have been proposed and developed. The present study thus focuses on optimization and improvement of the porous channel structure of P407/CMCs matrix to reduce the obstacle of matrix formulation for transport of targeted drugs, thereby increasing the concentration-dependent driving force and promoting transdermal diffusivity. The proposal was developed on the basis of close-packed array of P407 spherical micelles [22,36]. Lam and co-workers [20] observed that P407 micelles are spherical in shape and exhibit a core-shell structure, i.e., consisting of a hydrophobic PPO core and a hydrophilic shell, using cryogenic transmission electron microscopy (cryo-TEM) technique. The creation of 3D cubic lattice resulting from close-packed array of P407 spherical micelles is accountable for the formation of P407 gel, which is an endothermic process [18,22,36]. The close packing of P407 spherical micelles thus makes the formation of pore channels possible (Figure 1), which may serve as both a reservoir for hydrophilic drugs and a passageway for transport of targeted drugs. FE-SEM observation for the sectional view of pore channels (Figure 1) confirms that such porous matrix may provide a potential approach to optimizing and improving existing transdermal drug delivery systems to achieve higher drug permeation and bioavailability.

The viscosity behavior of drug carrier matrix is of high importance for diffusion and transport of targeted drugs. Therefore, viscosity properties of P407/CMCs composite hydrogel are first discussed. As shown in Figure 2, as expected, the complex viscosity of P407 matrix was typically concentration-dependent. PC200 showed medium viscosity and gelation transition temperature, and thus was selected for further investigation. The addition of CMCs was found to significantly increase viscosity of PC200, but slightly decrease the gelation transition temperature. The reason for this situation can be inferred from molecular interaction [37]. Hydrophilic CMCs interact with hydrophilic PEO chains via covalent bond after mixing with P407 solution and are thus located in the outer aqueous region instead of the hydrophobic interior of P407 spherical micelles. This means that the effective hydrophilic chains are increased, thereby causing an increase in bulk viscosity and a decrease in the gelation transition temperature.

Next, we investigated the pore shape and size distribution of P407 matrix with varying concentrations, qualitatively as well as quantitatively, as shown in Figure 3 and Table 1. Likewise, the pore morphology and size distribution are closely related to the P407 matrix concentration. The most probable pore size for PC160 and PC200 was comparable, while this figure for PC240 saw a sharp decline, at around 0.689 μm (Table 1). This could be better interpreted by the ordered micellar structure for P407 solution. Essentially, P407 is a non-ionic macromolecular surfactant agent. At low concentration of solutions, P407 molecules exist predominantly in monomeric form, i.e., unimers. As the concentration of P407 increases above the critical micellar concentration, thermodynamically stable micelles are formed [38]. Though the micelle size is independent of P407 concentration, the spherical micelles become compact with increasing concentration [38,39,40]. This provides a reason why the pore size of PC240 was much smaller than that of PC160 and PC200. It should be noted that pore sizes for the three specimens show a broad distribution, consistent with the low pore number fractions (Table 1).

The effect of the presence of CMCs on the porous structure of P407 matrix was further comparatively studied (Figure 4 and Table 1). It can be clearly seen that the porous structure of P407 was significantly changed on adding CMCs. The pore size distribution for both PC202 and PC204 was close to normal distribution. Moreover, the pore number fraction was increased around tenfold, both for PC202 and PC204, compared to PC200. The total surface area for both also saw an appreciable increase. This suggests that the porous structure of P407 matrix was markedly optimized and improved with the presence of CMCs. The reason is similar to the analysis for viscosity behavior, due to the prolonged effective hydrophilic chains.

The percutaneous diffusivity behavior for various P407/CMCs composite matrix is shown in Figure 5. For the convenience of discussion, various P407/CMCs hydrogels were grouped and shown in the histograms (Figure 6). Figure 6a presents P407 samples with different concentrations, i.e., PC160, PC200 and PC240. Obviously, P407 concentration was inversely proportional to the drug transdermal penetration. This reveals that PC160 had the highest drug transdermal level, whereas the lowest percentage of drug penetrated through PC240 across the skin, which was confirmed by their apparent permeability coefficients (*P*_app_ value) (Figure 6a). This phenomenon primarily stems from the fact that high viscosity of P407 matrix retards the diffusional release of drug. Consequently, the sample with the highest viscosity (PC240) resulted in the lowest *P*_app_ value. On the other hand, this situation was probably associated with the porous channel structure of P407 matrix. PC160 shows the largest pore size while this figure for PC240 is the smallest (Table 1). Therefore, the diffusion and transport of model drug through PC240 encountered the biggest resistance, whereas PC160 had the least. This could be the cause why PC240 has the lowest *P*_app_ value while PC160 shows the highest proportion of model drug having permeated across the skin.

Next, looking at P407/CMCs composite hydrogels (Figure 5), the rates of diffusion of drug from PC202 and PC204 were faster than PC200, indicating that the presence of CMCs enhanced the permeability of GA through the porcine skin. This is further validated by the *P*_app_ value in Figure 6b. However, in comparison with the complex viscosity (Figure 6b), this figure for PC204 is the highest while PC200 shows the lowest. This led to an incomprehensible contradiction because the bulk viscosity of P407 matrix was increased with the addition of CMCs, and the permeation proportion of model drug was also enhanced. This could not be ascribed to the penetration enhancing effect of CMCs, albeit chemical penetration enhancers are normally used to promote the diffusion of drug through the skin by overcoming the barrier function of stratum corneum [1]. Our previous work has demonstrated that CMCs have no permeation enhancing ability [28]. Consequently, the major reason for this factor primarily lies in the improved porous structure of P407 hydrogel matrix after the addition of CMCs. Although there was no significant difference (*p* > 0.05) in the most probable pore size among the three specimens, the pore number fractions for both PC202 and PC204 were increased over tenfold compared to PC200 (Table 1). This clearly reveals that the presence of CMCs improves the porous structure of P407 matrix. Accordingly, the diffusional release of model drug within PC202 and PC204 encountered much fewer obstacles and thus shows a higher permeation level across the skin. This also explains why PC204 saw a higher permeability level than PC202.

Additionally, it is noteworthy that the highest *P*_app_ value is seen in PC204, whereas PC240 shows the lowest permeability percentage for the model drug (Figure 6c). However, the complex viscosity for both was comparable. This further confirms that the presence of CMCs facilitated the permeability of drug across the skin. Clearly, the dominating factor contributing to this situation can be ascribed to the improved porous channel structure with the presence of CMCs, i.e., higher pore number fraction and approximate normal distribution. With the improved porous structure of P407/CMCs hydrogel matrix, the diffusion and transport of hydrophilic drugs thus become easier and more accessible, thereby facilitating the formation of drug concentration-gradient driving force and promoting the drug transdermal permeability (Figure 1).

**Figure 7:**
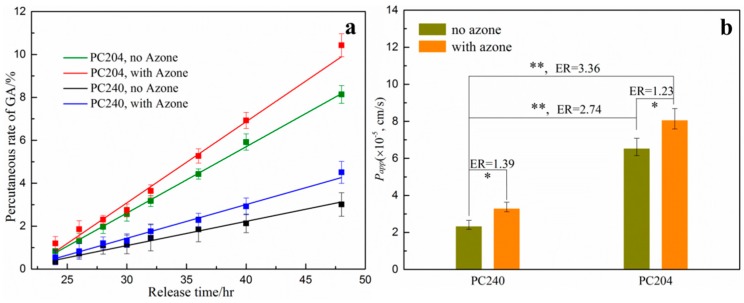
(**a**) In vitro cumulative diffusional release of GA penetrated through porcine ear skin from PC204 and PC240 hydrogel matrix with or without azone (*n* = 3); and (**b**) the apparent permeability coefficient (*P*_app_) and enhancement ratio (ER) (* *p* < 0.05; ** *p* < 0.01; HPLC mobile phase: acetonitrile and double distilled water/phosphoric acid (99.0/1.0, *v*/*v*), flow rate: 1.0 mL/min; the data for PC204 without azone extracted from [24]).

In order to further investigate the percutaneous behavior of model drug from the various P407/CMCs composite matrix, azone, a commonly used chemical penetration enhancer, was also employed and formulated within the matrix (Figure 7). PC240 and PC204 were selected as the carrier matrix due to the equivalent viscosity. As shown in Figure 7, drug permeability for PC240 was slightly increased with the presence of azone (*ER* = 1.39, *p* < 0.05). A similar situation was also seen in PC204 (*ER* = 1.23, *p* < 0.05). This suggests that the permeation behavior of model drug across the skin can be appreciably increased with the aid of azone. However, it is worth noting that, the permeability enhancement for the PC240 with azone was still far lower than that of PC204 without azone, notwithstanding that viscosity for both was almost equal. The primary cause of this phenomenon is the improved porous structure of PC204. This provides the strongest evidence for our proposal that the permeability behavior was considerably enhanced by optimizing and reconstructing the porous structure of P407 matrix through the addition of CMCs. Consequently, it can be concluded that, with the improved and optimized porous structure of P407/CMCs composite matrix, the transdermal permeability behavior can be further enhanced with the aid of penetration enhancers.

In summary, the presence of CMCs was found to optimize and improve the porous structure of P407 hydrogel matrix; such porous channel structure facilitates the percutaneous diffusional behavior of the model drug. Therefore, P407/CMCs composite hydrogel matrix with abundant porous structure may be an ideal drug carrier to load hydrophilic drugs for transdermal administration, avoiding or reducing the use of any chemical enhancers. Though the present study employed a small molecule model drug (GA), we believe that the porous P407/CMCs hydrogel matrix may also provide a feasible and promising way to load macromolecular drugs for transdermal application, especially with the aid of chemical penetration enhancers. Additionally, another advantage for this drug matrix is the controllable drug loading, simply by controlling the concentration of drugs when fabricating drug loaded hydrogel matrix.

## 5. Conclusions

This study has developed a novel improved porous P407/CMCs hydrogel drug carrier for transdermal purposes, avoiding the use of chemical enhancers. It was shown that the addition of CMCs noticeably improves the porous structure of P407 matrix and the transdermal permeability behavior of loaded drug can be markedly increased. The improvement and optimization of the porous channel structure of P407/CMCs hydrogel matrix are accountable for the increased drug diffusivity within the matrix, leading to an increase in the drug concentration-gradient driving force. Accordingly, the percutaneous behavior of targeted drugs is improved. This also provides a feasible and promising strategy to load macromolecular drugs, such as vaccines and peptides, etc., for transdermal application with a high bioavailability, simply by optimizing the microstructure of the drug carrier.

## Figures and Tables

**Table 1:** Mercury intrusion porosimetry analysis results.

Sample	PC160	PC200	PC240	PC202	PC204
The most probable pore-size/μm	4.989	4.813	0.689	4.701	4.343
Total surface area (m^2^/g)	1.502	1.709	2.887	1.808	1.996
Pore number fraction	0.0177	0.0212	0.0211	0.213	0.277
